# A versatile and GMP-compliant bead-based IFN-γ potency assay for standardized quality control and release testing of CAR-T cell drug products

**DOI:** 10.1016/j.ijpx.2026.100559

**Published:** 2026-05-04

**Authors:** Robin Dennebos, Macarena González-Corrales, Maria Lysandrou, Nienke A.M. Smit, Yuzhu Qi, Tom van Meerten, Jos G.W. Kosterink, Gerwin Huls, Harm-Jan Lourens, Bahez Gareb, Edwin Bremer

**Affiliations:** a Department of Hematology, University Medical Center Groningen, University of Groningen, Groningen, the Netherlands; b Department of Clinical Pharmacy and Pharmacology, University Medical Center Groningen, University of Groningen, Groningen, the Netherlands

**Keywords:** Potency assay, CAR-T cells, IFN-γ release, Good manufacturing practices (GMP), Quality control testing

## Abstract

Potency is a critical specification for CAR-T cells and is typically determined by co-culture of CAR-T cells with target cell lines. However, cell line heterogeneity hampers standardization and can lead to analytical variation impacting potency test results. Further, both the assay and cell line maintenance are labor, time, and resource intensive. Here, we developed and validated a versatile, fully standardized, and GMP-compliant CAR-T cell potency assay. This assay utilizes antigen-loaded beads instead of target cell lines and was fully validated for CD19 and qualified for CD7 and HER2 CAR-T cells. Incubation of streptavidin beads with recombinant biotinylated antigen yielded a dose-dependent bead loading. Subsequent incubation of CAR-T cells with increasing amounts of antigen-loaded beads yielded a dose-dependent secretion of IFN-γ, whereas non-loaded or MOCK antigen-loaded beads did not significantly trigger IFN-γ secretion. Notably, as assessed for CD19 CAR-T cells, cryopreserved CAR-T cells yielded lower potency than fresh CAR-T cells and potency results correlated with the total amount of CAR-T cells in the test sample. Therefore, the assay was fully standardized using a fixed amount of 50 k CAR-T cells per test, a fixed amount of antigen loaded onto the beads (1 pg/bead), and a fixed amount of 500 k antigen-loaded beads. A quantitative and statistically substantiated potency threshold for batch release was established for fresh as well as cryopreserved CAR-T cell drug products This fully standardized assay protocol and validation strategy provides a facile potency assay for CAR-T cell drug products that can be implemented for essentially any antigen of interest.

## Highlights

• Bead-based potency assays are versatile and do not require cell lines.
• CD19, CD7 and HER2 CAR-T cell potency assays are developed, demonstrating versatility.
• A validated and GMP-compliant bead-based potency assay for CAR-T cells is presented.
• Statistically substantiated potency acceptance criteria for batch release is presented.
• The bead-based assay does not require cell lines and strongly reduces labor burden.

## Graphical abstract

**Figure:**
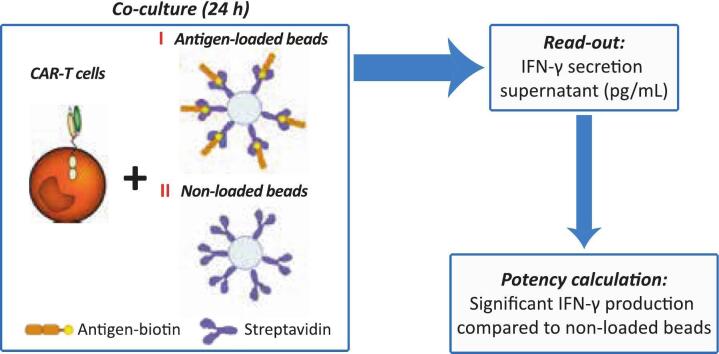

## 1 Introduction

Chimeric Antigen Receptor (CAR)-T cells have emerged as a key breakthrough in the treatment of B-cell malignancies, with long term remission and cure in previously end-stage patients. These unprecedented responses have clearly transformed the clinical landscape, with several commercial CAR-T cell products being available as standard-of-care in the US and EU. This includes CAR-T cell products for the treatment of hematological cancers such as multiple myeloma (Munshi et al., 2021), mantle cell lymphoma (Wang et al., 2020), refractory large B-cell lymphoma (Neelapu et al., 2017; Abramson et al., 2020), B-cell precursor acute lymphoblastic leukemia (Shah et al., 2021), and follicular lymphoma (Jacobson et al., 2022). In addition, many academic institutes develop point-of-care decentralized manufacturing of clinical-grade CAR-T cells including for anti-CD19 (Castella et al., 2019; Palani et al., 2023; Ortiz-Maldonado et al., 2022; Maschan et al., 2021), anti-CD20 (Lock et al., 2017), anti-BCMA (Asherie et al., 2022; Oliver-Caldés et al., 2023) therapy. Further, the field of CAR-T cells is rapidly evolving and moving into emerging applications in solid tumors, autoimmune disorders, and infectious diseases (Du et al., 2025; Yang et al., 2025).

CAR-T cells are classified as gene therapy medicinal products by the FDA (U.S. Food and Drug Administration, 2024) and EMA (Committee for Advanced Therapies (CAT), 2020) and should be manufactured, quality control (QC) tested, and released for human use under good manufacturing practice (GMP) conditions. Various QC tests are performed to ensure that the CAR-T cell product complies with predefined specifications that warrant product quality and safety. In this respect, FDA as well as EMA guidelines state that a potency assay should be performed as part of the QC testing regimen for CAR-T cells. Potency is defined as a quantitative measure of biological activity that is linked to the relevant biological properties and the claimed mechanism of action. Of note, patient (e.g., pretreatment and tumor burden) and CAR-T cell characteristics (e.g., fitness, phenotype, and persistence) determine the in vivo efficacy of CAR-T cell therapy in a complex interplay of multiple factors (Yoo et al., 2025; Tao et al., 2024). Thus, the objective of the potency assay is to demonstrate the functionality of the manufactured CAR-T cells instead of serving as a prognostic marker for the efficacy of CAR-T cell therapy. (U.S. Food and Drug Administration, 2024; Committee for Advanced Therapies (CAT), 2020).

CAR-T cell potency for drug product release is typically demonstrated with individualized and tailored in vitro assays, which are accepted by regulators for routine and GMP-compliant potency testing during batch release (U.S. Food and Drug Administration, 2024; Committee for Advanced Therapies (CAT), 2020). Although multiple assays can be utilized for such potency testing, the general principle is the same, namely measuring CAR-T cell activation upon target-antigen exposure, such as cytokine secretion or cytotoxicity measured through degranulation, chromium (^51^Cr) release, impedance-based, lactate dehydrogenase, or bioluminescence potency assays (Kiesgen et al., 2021). Besides the technical limitations of some of these assays such as expensive equipment (e.g., gamma scintillator counter or impedance analyzer), specialized staff, and radioactive nucleotides as well as waste handling, all these assays require target cell lines expressing the targeted antigen and luciferase in the case of the bioluminescence assay.

Unfortunately, cell line-based assays are inherently characterized by biological variability and heterogeneity. Cultured cells from a single cell line can diversify and change over time, which translates into cell line heterogeneity within and between laboratories. In this respect, altered cell morphology, doubling time, cellular motility and adhesion, karyotype, locus-specific methylation patterns, immunophenotype, pro- and anti-inflammatory cytokine secretion, and the expression of surface markers have been reported (Wilson et al., 2025; Sambuy et al., 2005; Putnik et al., 2015; Liu et al., 2019; Kasan et al., 2025; Delage et al., 2023; Ben-David et al., 2018). Even when the cells are cultured under controlled and standardized conditions, cell line evolution due to positive clonal selection is observed (Wilson et al., 2025; Sambuy et al., 2005; Putnik et al., 2015; Liu et al., 2019; Kasan et al., 2025; Delage et al., 2023; Ben-David et al., 2018).

As a consequence of cell line heterogeneity, validating a cell-based potency assay that yields reproducible test results during validation, but also after validation and over an extended period of time is challenging. For example, in case the expression of the target antigen and/or cytokine secretion profile changes over time, CAR-T cell activation during the assay may be impacted and, therefore, the potency test result. Furthermore, cell line heterogeneity between laboratories may also result in irreproducible test of validation results (i.e., impacting precision). Consequently, assay performance may not be comparable in time or between laboratories, which can affect CAR-T cell comparability study results (Delage et al., 2023). The latter is especially important for point-of-care CAR-T cell manufacturing at multiple manufacturing sites.

In addition, for cell-based assays both target-antigen- and non-target-antigen-expressing cell lines (as negative control) should be readily available in culture during potency testing. However, there are many novel CAR-T cell products being developed against a range of target antigens (e.g., CD23 (Giordano Attianese et al., 2011), CD32b (Wang et al., 2021), CD70 (Shaffer et al., 2011), CD72 (Nix et al., 2021), CD133 (Bueno et al., 2019), TSLPR (Qin et al., 2015), and Siglec-6 (Kovalovsky et al., 2021)) as well as bi- or multispecific CAR-T cells (e.g. CD19xCD20 or CD19xCD20xCD22) (Kulkarni et al., 2025). As CAR-T cell manufacturers can manufacture different CAR-T cell products at one manufacturing site, bespoke potency testing for each CAR-T cell product will necessitate the development, validation, use and maintenance of multiple cell lines expressing the target antigen and their continuous culture. This is labor intensive, time consuming, error-prone, and resource intensive. Therefore, a fully standardized, GMP-compliant, and versatile CAR-T cell potency assay in which the targeted antigen can be varied to facilitate potency testing of current as well as future CAR-T cell products is desired. However, such potency assays are currently unavailable.

The objective of this study was to develop and validate a versatile, rapid, fully standardized, and GMP-compliant CAR-T cell potency assay that does not require cell lines. The developed potency assay utilized standardized antigen-loaded beads with which CAR-T cells were incubated for precisely calibrated antigen-specific activation upon which IFN-γ secretion was analyzed. The potency assay was fully validated and characterized for CD19 CAR-T cells. As a proof-of-concept and to substantiate the versatility of the assay, the potency assay was further adapted to HER2 and CD7 CAR-T cell potency testing. The presented potency assay, validation strategy, and acceptance criteria can be used for the standardized and harmonized development of a potency test for CAR-T cells targeting essentially any antigen of interest.

## 2 Material and methods

### 2.1 T cell culture and transduction with CAR constructs

CD19 and CD7 CAR T cells were generated using the CliniMACS Prodigy (Miltenyi Biotec, Leiden, The Netherlands). Apheresis material was collected and incubated overnight at 4 °C. After overnight incubation apheresis material was transferred to the CliniMACS Prodigy® TS 520 (Miltenyi Biotec) connected to the CliniMACS Prodigy for automated cell culture and further processing. CD4/CD8 selection of T cells was performed using the CliniMACS Prodigy, CliniMACS PBS/EDTA buffer (Miltenyi Biotec) supplemented with 0.5% human serum albumin (Prothya Biosolutions Netherlands B.V., Amsterdam, The Netherlands), CliniMACS® CD4 Reagent CR/GMP (Miltenyi Biotec) and CliniMACS® CD8 Reagent CR/GMP (Miltenyi Biotec). After CD4/CD8 sorting, 200 × 10^6^ viable CD3 positive cells were seeded into the cultivation chamber. CD4/CD8-selected T cells were cultured in TexMACS™ GMP Medium (Miltenyi Biotec) supplemented with 25 μg MACS® GMP Recombinant Human IL-7 (Miltenyi Biotec) and 25 μg MACS® GMP Recombinant Human IL-15 (Miltenyi Biotec). CD4/CD8 selected T cells were activated on the day of seeding in the CliniMACS Prodigy using MACS® GMP T Cell TransAct™ Large Scale CR/GMP (Miltenyi Biotec). 16 h after activation, CD4/CD8-selected T cells were transduced with lentivirus encoding CD19 CAR or CD7 CAR. CAR-T cells were collected on day 6 or day 11 with culture wash, media exchange and shaker steps performed automatically by the CliniMACS Prodigy.

For HER2 CAR T cells, CD4/CD8 T cells were isolated from PBMCs obtained from buffy coats using CD4 and CD8 MicroBeads (Miltenyi Biotec) and the MACS separator (Miltenyi Biotec). CD4/CD8-selected T cells were activated using CD3/CD28 Dynabeads™ Human T-activator (Thermo Scientific, Waltham, MA, USA) 24 h prior to lentiviral transduction. Specifically, T cells were seeded at a density of 1 × 10^6^/mL in RPMI 1640 supplemented with 20% FBS (Sigma-Aldrich, St. Louis, MO, USA), 6000 IU/mL IL-2 (UMCG pharmacy, Groningen, The Netherlands) and transduced with lentivirus encoding HER2 CAR. 72 h after transduction, T cells were washed with PBS supplemented with 2% FBS and transduction efficiency was measured using a CytoFLEX flow cytometer (Beckman Coulter Brea, CA, USA).

For CD7 and HER2 CART-T cells, primary T cells were obtained from buffy coat samples originating from healthy donors (Sanquin, The Netherlands; agreement number NVT0465.01). For CD19 CAR-T cells, apheresis material from relapsed/refractor large diffuse B-cell lymphoma patients participating in a clinical trial (EUCT protocol code 2024–511979–15-00) was used to generate the CAR-T cells after informed consent was obtained. This clinical trial was approved by the medical ethical committee and was performed in accordance with the declaration of Helsinki.

### 2.2 Bead loading and standard curve

Dynabeads™ M-280 Streptavidin (Invitrogen™, Thermo Scientific, Waltham, MA, USA) were loaded with CD19 CAR Detection Reagent, human, Biotin (Miltenyi Biotec), Biotinylated Recombinant Human CD7 (carrier-free) (Biolegend, California, USA), Recombinant human ErbB2/HER2 (biotinylated) protein (Abcam, Cambridge, UK) or incubated with 1× phosphate buffered saline (PBS) as a non-loaded control. Beads were counted using a MacsQuant Analyzer 10 (Miltenyi Biotec) and 500 × 10^3^ were washed and resuspended in PBS. Removal of washing solutions was perfomed using a Magnarack (Invitrogen™, Thermo Scientific) or DynaMag™-2 (Invitrogen™, Thermo Scientific) magnetic rack. Loading of beads was achieved by incubating the beads with the biotinylated protein for 30 min in the dark at room temperature (RT). For the standard curve, 500 × 10^3^ beads were loaded with different amounts of biotinylated protein by serial dilution in PBS. Antigen loading per bead was determined to be optimal at 1125 pg for CD19 CAR Detection Reagent, 2217 pg for biotinylated recombinant human CD7 and 1,25 pg for recombinant human ErbB2/HER2. After loading beads were washed twice with PBS followed by a washing step with RPMI 1640 supplemented with 10% FBS. After washing, beads were resuspended in RPMI 1640 supplemented with 10% FBS and counted. RPMI 1640 supplemented with 10% FBS was added to the loaded beads to achieve a final bead concentration of 5 × 10^6^ per mL.

### 2.3 Antibodies and Flow Cytometry

Phycoerythrin (PE)-conjugated CD19, allophycocyanin (APC)-conjugated CD7 and fluorescein isothiocyanate (FITC)-conjugated HER2 antibodies (Biolegend) were used to detect the presence of the antigen on cell lines and the presence of the CAR detection proteins on beads. CD3 antibody (Biolegend) was used to select for CD3 T cells from the CD4/CD8-selected T cells. Flow cytometry data was obtained on the MacsQuant Analyzer 10 (Miltenyi Biotec).

### 2.4 Linearity, range and IFN-γ potency assay

To determine linearity and range of beads, a concentration range of 1 × 10^3^ to 500 × 10^3^ non-loaded, target antigen-loaded (CD19, CD7 or HER2 protein) or MOCK-loaded (CD19 or CD7 protein) beads (1 × 10^3^/ 5 × 10^3^/ 10 × 10^3^/ 50 × 10^3^/ 100 × 10^3^/ 500 × 10^3^ beads) was co-cultured with 50 × 10^3^ T cells containing CAR-T cells. Beads were loaded with the optimal concentration of protein determined in the standard curve. After loading, beads were diluted with RPMI supplemented with 10% FBS and co-cultured with CAR-T cells in a Nunc™ MicroWell™ 96-Well, Nunclon Delta-Treated, Flat-Bottom Microplate (Thermo Scientific) in different effector:target (E:T) ratios for 24 h at 37 °C. CAR-T cells were incubated for 24 h in order to ensure that maximal activation has occurred and the time required for induction of production and secretion of IFN-γ for individual CAR-T cell drug products is not a confounding factor in the assay (Patel et al., 2024). After incubation, samples were harvested and centrifuged at 450 *g* for 5 min, supernatant was recovered and IFN-γ was measured. To test the potency of CAR-T cells, 50 × 10^3^ T cells containing CAR-T cells were co-cultured with 500 × 10^3^ beads (non-loaded, target antigen-loaded or MOCK-loaded) for 24 h at 37 °C. Beads were prepared as explained in paragraph 2.2. CAR-T cells were washed with PBS and resuspended in RPMI supplemented with 10% FBS. After 24 h, the plate was centrifuged at 450 *g* for 5 min and supernatant was recovered. IFN-γ levels in collected supernatants were either measured directly or stored at −20 °C and measured at a later timepoint. IFN-γ levels were measured using the Ella (Ella™ Automated ELISA; Bio-Techne) using Simple Plex Human IFN-gamma (3rd Gen) Cartridges (Bio-Techne). The potency test result was determined by subtracting the non-loaded bead potency test value (negative control) from the antigen-loaded bead potency test value obtained after 24 h of co-culture with CAR-T cells.

### 2.5 Stability study CD19-loaded beads

Stability of CD19-loaded beads was determined by loading streptavidin beads with CD19 CAR Detection Reagent, human, Biotin (1125 pg/bead) followed by 2,4 and 8 weeks storage at 4 °C (setpoint) in a temperature-monitored refrigerator (2–8 °C). Beads were prepared as described in Section 2.2. At 0, 2, 4 and 8 weeks bead loading was determined by flow cytometry using PE-conjugated CD19, and an IFN-γ potency assay was performed by co-culturing stored non-loaded and CD19-loaded beads with 50 × 10^3^ CAR-T cell drug product cells for 24 h at 37 °C. After co-culture supernatant was collected and IFN-γ levels were measured as described in Section 2.4.

### 2.6 Statistics and calculations

Combined potency test results of CAR-T cells originating from multiple donors were presented as median ± IQR since these results were not normally distributed and skewed, presumable due to donor-to-donor variability.

Assay precision was calculated using the coefficient of variation (CV), i.e. the ratio of the obtained standard deviation (s.d.) to the mean of the test results.

For the potency threshold, the Wilcoxon signed-rank test (paired non-parametric data) was used to test for a statistical significant increase in IFN-γ compared to non-loaded beads as the control group (negative control). Since the control group was used twice during statistical testing, the Bonferroni correction was used, resulting in a significance level of 0.05/2 = 0.025. Statistical testing was performed in SPSS 28 (IBM, New York, USA).

## 3 Results and discussion

### 3.1 Rationale and validation strategy of the potency assay

**Fig. 1:**
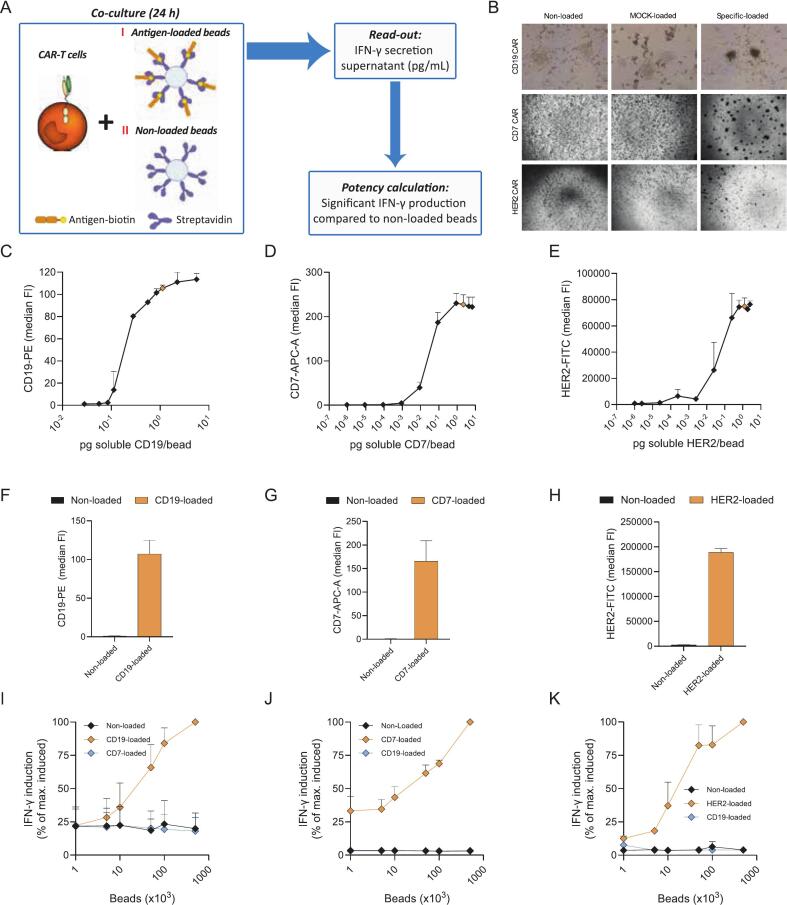
Potency assay antigen loading, specificity, and dose-response relationship. (A) Graphical illustration of the bead-based potency assay. (B) Microscopic images (×200 magnification for CD19, ×100 for CD7 and HER2 CAR-T cells) taken of different conditions (non-loaded, MOCK-loaded or target antigen-loaded) of the bead-based potency assay after 24 h of CAR-T cell and beads co-culture (beads loaded with 1.13 pg/bead for CD19, 2.23 pg/bead for CD7 and 1.25 pg/bead for HER2). (C-E) Dose-dependent target antigen loading curves of CD19 (C), CD7 (D) and HER2 (E) biotinylated proteins on streptavidin-coated beads. Median fluorescence intensity (MFI) is plotted against increasing target antigen concentrations. Orange points indicate the antigen amounts selected for further experiments (1.13 pg/bead for CD19, 2.22 pg/bead for CD7 and 1.25 pg/bead for HER2). Mean ± SD is shown (*n* = 3). (F—H) Flow cytometry analysis of non-loaded and target antigen-loaded beads stained with anti-CD19 (F), anti-CD7 (G) or anti-HER2 (H) antibodies to assess bead interference during antigen loading analysis. Mean ± SD is shown (*n* = 4–10). (I—K) Concentration dependent increase in CD19 (I), CD7 (J), and HER2 (K) CAR-T cell activation measured as IFN-γ secretion by co-incubation with increasing amount of antigen-loaded beads. Non-loaded and MOCK-loaded beads were included as negative controls. Values are shown as percentage of maximum induced of antigen-loaded beads. Mean ± SD is shown (*n* = 2–4).

The objective of this study was to develop a fully standardized, versatile, and GMP-compliant potency assay for quality control release testing of CAR-T cell drug products. For this purpose, a bead-based assay was designed in which a controlled amount of recombinant biotinylated antigen of interest (target antigen) was loaded onto streptavidin beads. Subsequently, these target antigen-loaded beads were used to specifically trigger CAR-T cell signaling activity (Fig. 1A).

The secretion of IFN-γ was selected as functional read-out in line with FDA, EMA, and ICH guidelines for potency testing (U.S. Food and Drug Administration, 2024; Committee for Advanced Therapies (CAT), 2020; The International Council for Harmonisation of Technical Requirements for Pharmaceuticals for Human Use (ICH), 1999). This assay does not require cell lines expressing the target antigen and the amount of antigen loaded onto the beads can be controlled and fixed, thereby, fully standardizing the assay and eliminating heterogeneity of CAR-T cell activation. Herewith, potency test result variability within and between laboratories can be minimized. Furthermore, any biotinylated antigen of interest can in theory be loaded onto the beads, which renders the assay versatile and compatible with potency testing of current and future CAR-T cell products. Although antigen-coated beads are already utilized in cell activation protocols and assays (Wang et al., 2024; Harari-Steinfeld et al., 2022), the application towards a fully standardized, validated, and GMP-compliant potency assay for quality control testing and batch release of CAR-T cell drug products has not yet been presented.

Since the bead-based potency assay is intended for potency testing of autologous CAR-T cell products, there will be inherent variability in the test samples that cannot be fully attributed to the manufacturing process or analytical test method due to donor variability. Furthermore, no international reference standard is available for validation purposes. Therefore, an in-house validation strategy and acceptance criteria were developed based on relevant guidelines for the development and validation of analytical methods and potency testing (Table 1) (The International Council for Harmonisation of Technical Requirements for Pharmaceuticals for Human Use (ICH), 1999; The International Council for Harmonisation of Technical Requirements for Pharmaceuticals for Human Use (ICH), 2023).

**Table 1:** Validation strategy and acceptance criteria for the bead-based potency assay.

Validation parameter	Acceptance criteria	
	CD19 CAR-T cells	HER2 or CD7 CAR-T cells
Assay versatility	Multiple target antigens (CD19, CD7, HER2) must be reproducibly loaded onto the beads in a concentration dependent and controlled manner	
Bead interference	Non-loaded beads must not interfere with antigen-loaded bead analysis to determine amount of antigen loaded onto the beads	
Concentration dependency	Concentration dependent increase in CAR-T cell activation by increasing amount of antigen-loaded beads	
Precision	Intra-assay precision (repeatability): <20% Intermediate precision: <25%	Intra-assay precision (repeatability): <20%
Specificity	Target antigen-loaded bead induce IFN-γ secretion in a dose-dependent manner; Non-loaded or MOCK-loaded bead do not induce significant IFN-γ secretion	
Potency threshold	Lowest target antigen-loaded bead concentration that activate all tested CAR-T cell batches and that induces statistically significant IFN-γ secretion of CAR-T cells compared to non-loaded beads as the negative control	Not tested

The assay was fully validated and characterized for CD19 CAR-T cells since multiple clinical-grade CD19 CAR-T cells are commercially and academically manufactured. To substantiate the versatility of the assay, the assay was further developed and qualified for two novel CAR-T cell products (i.e., HER2 and CD7 CAR-T cells) that are currently being developed in-house towards GMP-compliant manufacture for a phase I/II clinical trial.

### 3.2 Assay versatility, antigen loading, and bead interference

First, assay versatility was evaluated by loading of the three biotinylated target antigens CD19, CD7, and HER2 onto streptavidin beads followed by detection of the recombinant antigens using fluorescently labelled antibodies according to predefined validation acceptance criteria (Table 1). All three recombinant antigens were loaded in a concentration-dependent manner, with each antigen having a distinctive ED50 concentration as well as antigen saturation point beyond which no substantial increase in antigen loading was detected. For CD19, CD7, and HER2 the antigen saturation points were between 1 and 10 pg per bead for each target antigen (Fig. 1C-E). The loading intensity as defined by fluorescent intensity differed markedly between antigens, which could be due to antigen/biotin ratio as well as differences in affinity of the detection antibody and fluorophore conjugation and efficacy. Nevertheless, for each antigen a clear dose-dependent saturation curve was detected.

To standardize the assay protocol, a fixed amount of 500 k beads per test condition was selected paired with a fixed antigen load of 1.13 pg, 2.22 pg, and 1.25 pg antigen per bead for the CD19, CD7, and HER2, respectively, antigens. The selected amount of recombinant antigen corresponded to the respective antigen saturation point and was selected to eliminate any assay variability due to varying antigen loading efficiency (Fig. 1C-E). This fixed amount of fully antigen-loaded beads translates to a standardized CAR-T cell activation stimulus during the assay and is expected to eliminate test result variability such as arising from cell line heterogeneity or changes in the expression patterns of the target antigen over time (Wilson et al., 2025; Liu et al., 2019).

No background fluorescence was detected with non-loaded beads for any of the recombinant target antigens at these selected assay conditions, ensuring that the analytical response was solely due to the loaded target antigen (Fig. 1F-H). Therefore, the amount of loading of recombinant antigen onto beads was accurately assessed without interference.

These assay test results complied with the validation acceptance criteria (Table 1), thereby, demonstrating assay versatility and successful loading of beads with recombinant biotinylated antigens targeted by CAR-T cells.

### 3.3 Assay specificity

Next, the ability of target antigen-loaded beads to trigger CAR signaling was assessed. Significant clustering of CAR-T cells was detected upon incubation with target-antigen-loaded beads, but not with non-loaded beads or beads loaded with control antigen (MOCK), confirming antigen-specific activation (Fig. 1B). In line with this, incubation of CD19, CD7, and HER2 CAR-T cells with increasing amounts of target antigen-loaded beads yielded a clear dose-dependent secretion of IFN-γ for all three CAR-T cell products (Fig. 1I-K). In contrast, CAR-T cells did not significantly secrete IFN-γ even at the highest concentration of non-loaded or MOCK-loaded beads tested (Fig. 1I-K). Thus, the assay had high specificity for the target antigen of each respective CAR-T cell product, thereby, demonstrating assay specificity.

Of note, the dose-dependent increase in CAR-T cell activation by increasing amounts of antigen underscored the necessity to standardize the antigen amount to which the CAR-T cells were exposed during potency testing for reproducible assay results. Such reproducibility is considered impossible for cell-based potency assays due to cell line heterogeneity and changes in surface antigens over time, even for cell lines cultured in standardized conditions within a given laboratory (Wilson et al., 2025; Liu et al., 2019; Ben-David et al., 2018). In a worst-case scenario, fully functional and potent CAR-T cells may be less activated due to reduced expression of the target antigen by the cell line, thereby, possibly not complying with the potency acceptance criterion.

Taken together, the assay was proven to be target-antigen specific with a clear dose-dependent increase in IFN-γ secretion, thereby, complying with the assay validation acceptance criteria (Table 1).

### 3.4 Intra-assay precision

To qualify each potency assay, the repeatability was assessed by determining the intra-assay precision. The intra-assay precision of the CD19, CD7, and HER2 potency assays expressed as CV ranged between 2–13% (Table 2), 3–8% (Table 3), and 2–10% (Table 4), respectively. For all three CAR-T cell products, assay test results complied with the intra-assay precision acceptance criteria (Table 1), thereby, demonstrating assay repeatability.

**Table 2:** The intra-assay precision (repeatability) of the CD19 CAR-T cell potency assay. CD19 CAR-T cells were generated from patients' apheresis material.

CAR-T batch	Sample ID	IFN-γ secretion (pg/mL)	Mean IFN-γ secretion (pg/mL)	Standard deviation (pg/mL)	Intra-assay precision (CV%)
Batch 1	Sample 1	6273	6547	198	3
	Sample 2	6635			
	Sample 3	6733			
Batch 2	Sample 1	8542	8435	207	2
	Sample 2	8619			
	Sample 3	8146			
Batch 3	Sample 1	2355	2901	392	13
	Sample 2	3096			
	Sample 3	3253			
Batch 4	Sample 1	7810	8131	232	3
	Sample 2	8352			
	Sample 3	8231			
Batch 5	Sample 1	2679	2616	58	2
	Sample 2	2631			
	Sample 3	2539			

CV: coefficient of variation. ID: identification number.

**Table 3:** The intra-assay precision (repeatability) of the CD7 CAR-T cell potency assay. CD7 CAR-T cells were generated from buffy coat.

CAR-T batch	Sample ID	IFN-γ secretion (pg/mL)	Mean IFN-γ secretion (pg/mL)	Standard deviation (pg/mL)	Intra-assay precision (CV%)
Batch 1	Sample 1	19,460	17,606	1317	7
	Sample 2	16,524			
	Sample 3	16,834			
Batch 2	Sample 1	51,238	51,692	1731	3
	Sample 2	54,002			
	Sample 3	49,835			
Batch 3	Sample 1	25,044	25,736	1991	8
	Sample 2	23,718			
	Sample 3	28,446			

CV: coefficient of variation. ID: identification number.

**Table 4:** The intra-assay precision (repeatability) of the HER2 CAR-T cell potency assay. HER2 CAR-T cells were generated from buffy coat.

CAR-T batch	Sample ID	IFN-γ secretion (pg/mL)	Mean IFN-γ secretion (pg/mL)	Standard deviation (pg/mL)	Intra-assay precision (CV%)
Batch 1	Sample 1	1491	1378	133	10
	Sample 2	1192			
	Sample 3	1451			
Batch 2	Sample 1	919	866	38	4
	Sample 2	846			
	Sample 3	832			
Batch 3	Sample 1	801	787	18	2
	Sample 2	799			
	Sample 3	761			

CV: coefficient of variation. ID: identification number.

### 3.5 Stability study of CD19-loaded beads

**Fig. 2:**
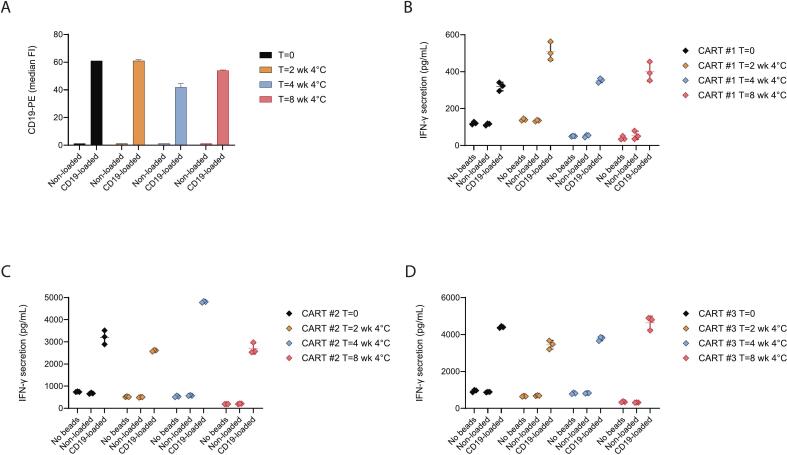
Bead stability study of CD19 loading and IFN-γ secretion of CAR-T cell drug product batches. (A) CD19- or non-loaded beads were freshly prepared and analyzed (*T* = 0) after which they were stored at 4 °C for 2, 4, and 8 weeks. Mean ± SD is shown (n = 4). (B/C/D) IFN-γ secretion upon antigen-specific activation of three CD19 CAR-T cell drug product batches generated from patients' apheresis material using beads stored at 4 °C for 2, 4, and 8 weeks. Mean ± SD is shown (*n* = 3 patients).

An 8-week stability study was conducted to assess the stability of CD19-loaded beads. On *T* = 0 weeks, CD19 bead loading was demonstrated by the detection of CD19 compared to non-loaded beads (Fig. 2A). Co-incubation with these loaded beads significantly activated CD19 CAR-T cells compared to non-loaded beads, as demonstrated by the increased IFN-γ secretion of three CAR-T cell batches. CD19 bead loading was detected up to 8 weeks of storage at 4 °C. Furthermore, co-incubation with these CD19-loaded beads stored at 4 °C for 2, 4 and 8 weeks significantly activated all CAR-T cell batches that were tested compared to non-loaded beads (Fig. 2B-D). Notably, compared to freshly prepared CD19-loaded beads (T = 0 weeks), IFN-γ secretion by activated CAR-T cells was comparable and not significantly reduced on every measured timepoint, indicating that the stored CD19-loaded beads were able to significantly activate CD19 CAR-T cells for up to 8 weeks. Therefore, CD19-loaded beads were considered stable for up to 8 weeks stored at 4 °C, which enables the preparation of a stock solution of CD19-loaded beads that can be stored refrigerated and used for multiple days.

### 3.6 CD19 CAR-T cell potency assay characterization and optimization

**Fig. 3:**
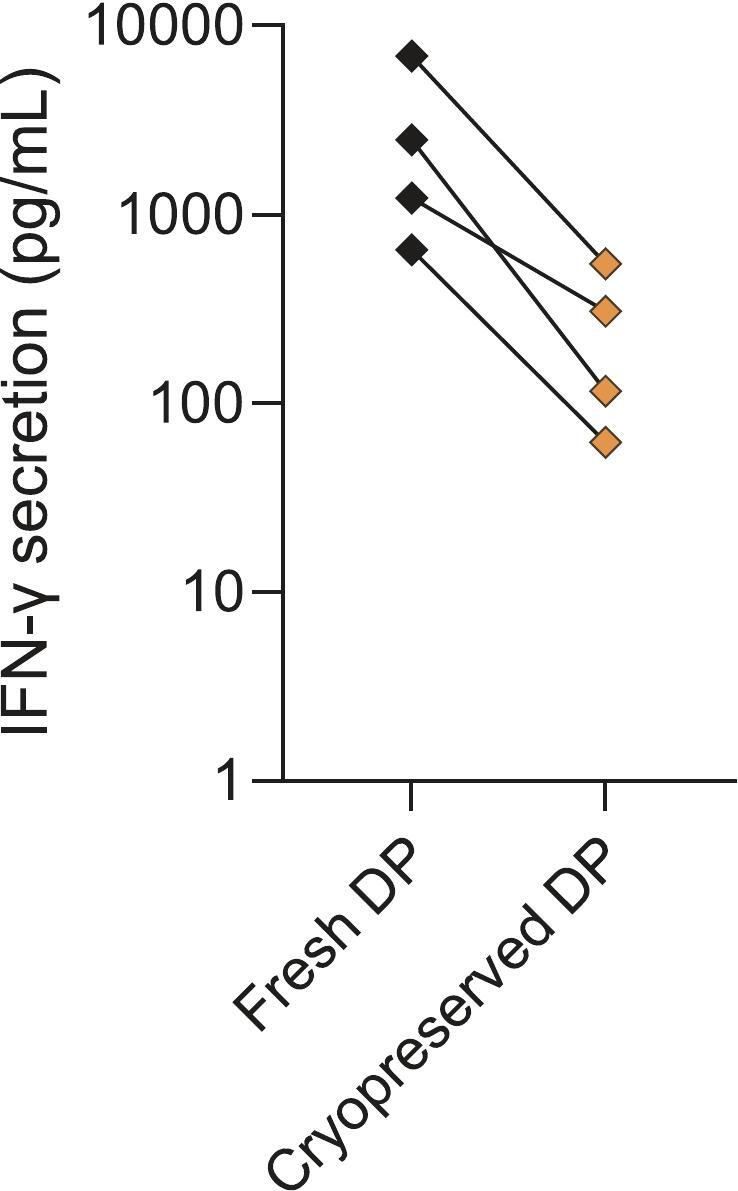
Potency assay test results of fresh versus cryopreserved CD19 CAR-T cell drug products (DP) generated from patients' apheresis material. Lines indicate paired data derived from samples from the same patient (*n* = 4 patients).

The CD19 potency assay was further characterized and optimized before being considered fully standardized and validated for routine GMP QC release testing. Specifically, during initial validation experiments a fixed amount of 50 k cryopreserved drug product cells were used. However, the potency test results of cryopreserved CAR-T cells proved to be lower than those of fresh CAR-T cells (Fig. 3). Thus, cryopreservation impacted the potency test results. Further, even when CAR-T cell batches are manufactured with a fully standardized and validated manufacturing process the transduction efficiency varies between donors. Indeed, the transduction efficiency of initially tested cryopreserved CD19 CAR-T cell drug products ranged from 65%–73%. Consequently, the number of CAR-T cells in the assay test sample varied from 32,500–36,500 CAR-positive cells.

**Fig. 4:**
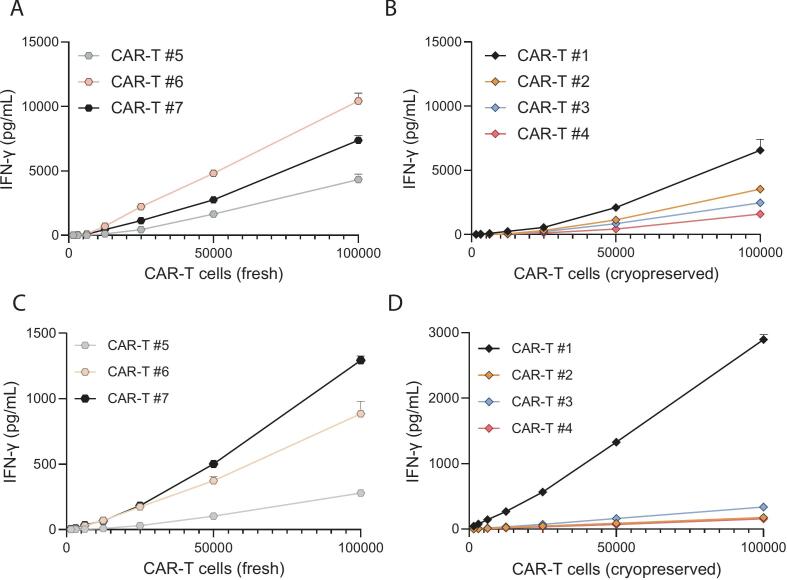
IFN-γ secretion and potency test results are impacted by amount of CD19 CAR-T cell present in the potency test sample. IFN-γ secretion upon target antigen activation is positively correlated with amount of activated fresh (A) as well as cryopreserved (B) CD19 CAR-T cells in the potency assay test samples (1563–100,000 CAR-T cells incubated with 500 k antigen-loaded beads). Baseline IFN-γ production of non-activated fresh (C) as well as cryopreserved CD19 CAR-T cells (D) is also positively correlated with amount of non-activated CAR-T cells (1563–100,000 CAR-T cells incubated with 500 k non-loaded beads as negative control). Mean ± SD of three technical replicates per patient is shown (*n* = 3–4 patients).

**Fig. 5:**
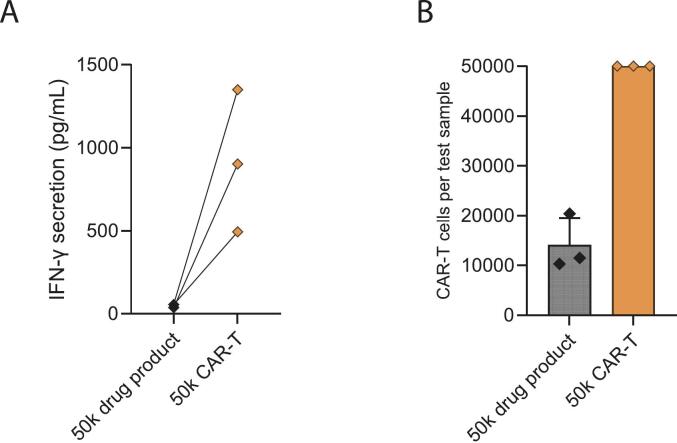
Comparative potency study of three fresh CAR-T cell batches tested with either 50 k drug product cells or 50 k CAR-T cells generated from patients' apheresis material. (A) IFN-γ secretion of 50 k drug product cells compared to 50 k CAR-T cells. Lines indicate paired data derived from samples from the same patient (*n* = 3 patients). (B) Comparison of amount of CAR-T cells present in the potency test sample during the IFN-γ potency assay between 50 k drug product cells (not corrected for transduction efficiency) or 50 k CAR-T cells. Mean ± SD of three technical replicates per patient is shown (*n* = 3 patients).

To assess whether and to what degree the amount of fresh or cryopreserved CAR-T cells present in the potency assay test sample could impact the test result, IFN-γ secretion in relation to increasing numbers of activated and non-activated CAR-T cells (with a range of 1563–100,000 CAR-T cells) were analyzed. Importantly, upon specific antigen-stimulation of CAR-T cells, a strong positive correlation with the amount of IFN-γ was detected with increasing numbers of fresh (Fig. 4A) and cryopreserved (Fig. 4B) CAR-T cells. In line with expectation, baseline IFN-γ secretion also positively correlated with the amount of fresh (Fig. 4C) and cryopreserved (Fig. 4D) non-activated CAR-T cells in the potency test sample. Taken together, these findings confirmed that the potency test results were impacted both by cryopreservation and by using a fixed number of total drug product cells without accounting for the transduction efficiency. Consequently, the potency test results of CAR-T cell batches with low transduction efficiency may be lower and possibly result in out-of-specification test results, despite containing fully functional and potent CAR-T cells. This was exemplified by a comparative study, in which three fresh CAR-T cell batches with either 50 k drug product cells or 50 k CAR-T cells were tested for potency (Fig. 5), with the potency test results for 50 k drug product cells (median: 55; range: 38–59 pg/mL) being significantly lower than for 50 k CAR-T cells (median: 903; range: 494–1350 pg/mL).

To address this test sample variability, the assay protocol was optimized and standardized to 50 k CAR-T cells. Therefore, the amount of beads, the amount of antigen loading per bead, and the number of CAR-T cells in the test sample were standardized, yielding a fixed amount of CAR-T cells and antigen-specific activation stimulus during each potency test.

### 3.7 Optimized CD19 CAR-T cells potency test precision

After assay optimization, additional precision experiments were performed to ensure that the assay complied with the validation acceptance criteria. First, intra-assay precision (repeatability) of the assay was assessed for fresh and cryopreserved CD19 CAR-T cells, which ranged from 1–6% and 2–18%, respectively (Table 5).

**Table 5:** The intra-assay precision (repeatability) of fresh and cryopreserved (cryo) CD19 CAR-T cell batches. CD19 CAR-T cells were generated from patients' apheresis material.

Fresh CAR-T cells	Sample ID	IFN-γ secretion (pg/mL)	Mean IFN-γ secretion (pg/mL)	Standard deviation (pg/mL)	Intra-assay precision (CV%)
Batch 1	Sample 1	6271	6214	49	1
	Sample 2	6187			
	Sample 3	6185			
Batch 2	Sample 1	5349	5235	103	2
	Sample 2	5206			
	Sample 3	5149			
Batch 3	Sample 1	9551	9284	307	3
	Sample 2	8948			
	Sample 3	9353			
Batch 4	Sample 1	10,298	9727	539	6
	Sample 2	9226			
	Sample 3	9656			
Cryo CAR-T cells	Sample ID	IFN-γ secretion (pg/mL)	Mean IFN-γ secretion (pg/mL)	Standard deviation (pg/mL)	Intra-assay precision (CV%)
Batch 1	Sample 1	3445	3219	240	7
	Sample 2	3246			
	Sample 3	2967			
Batch 2	Sample 1	1319	1355	32	2
	Sample 2	1365			
	Sample 3	1380			
Batch 3	Sample 1	809	812	49	6
	Sample 2	863			
	Sample 3	765			
Batch 4	Sample 1	211	267	48	18
	Sample 2	298			
	Sample 3	291			

CV: coefficient of variation. ID: identification number.

Subsequently, the intra-assay (repeatability) as well as intermediate precision of the assay was assessed for cryopreserved CAR-T cells. Cryopreserved CAR-T cells were selected as the worst-case scenario since the potency test results of cryopreserved CAR-T cells were on average lower, but the test result variability were similar to the variability observed for fresh CAR-T cells (Fig. 3 and Table 5). Consequently, lower assay precision was observed in case cryopreserved CAR-T cells were tested for potency. These additional precision experiments demonstrated an intra-assay and intermediate precision of 2–18% and 6–21%, respectively (Table 6), thereby, complying with the validation acceptance criteria (Table 1).

**Table 6:** The intra-assay (repeatability) and intermediate precision of cryopreserved CD19 CAR-T cell potency assay. CD19 CAR-T cells were generated from patients' apheresis material.

IFN-γ secretion		Sample 1 (pg/mL)	Sample 2 (pg/mL)	Sample 3 (pg/mL)	Mean samples (pg/mL)	SD samples (pg/mL)	Intra-assay precision (CV%)	Mean operators (pg/mL)	SD operators (pg/mL)	Intermediate precision (CV%)
Batch 1	Operator 1	1206	1161	1308	1225	75	6	1429	190	13
	Operator 2	1667	1588	1795	1683	104	6			
	Operator 3	1360	1339	1436	1378	51	4			
Batch 2	Operator 1	5020	5401	5403	5275	221	4	5260	1091	21
	Operator 2	6410	6624	6732	6589	164	2			
	Operator 3	3993	3940	3817	3917	90	2			
Batch 3	Operator 1	3444	3246	2967	3219	240	7	3430	221	6
	Operator 2	3471	4050	3685	3735	293	8			
	Operator 3	3563	3147	3301	3337	210	6			
Batch 4	Operator 1	1319	1365	1380	1355	32	2	1173	181	15
	Operator 2	1232	1279	1200	1237	40	3			
	Operator 3	911	942	925	926	16	2			
Batch 5	Operator 1	809	863	765	812	49	6	885	65	7
	Operator 2	901	838	876	872	32	4			
	Operator 3	948	1035	928	970	57	6			
Batch 6	Operator 1	211	298	291	267	48	18	284	20	7
	Operator 2	263	282	274	273	10	3			
	Operator 3	292	346	295	311	30	10			

CV: coefficient of variation. SD: standard deviation.

### 3.8 Optimized CD19 CAR-T cells potency threshold

**Fig. 6:**
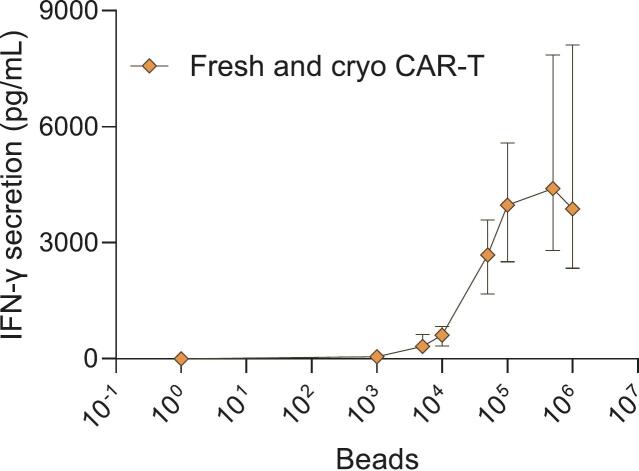
Combined dose dependent titration curve of 50 k fresh (*n* = 13 patients) and cryopreserved CD19 CAR-T cells (*n* = 5 patients) generated from patients' apheresis material. Median ± IQR is shown (*n* = 18 patients). The wide IQR indicates large patient-to-patient variability in the potency test result.

**Fig. 7:**
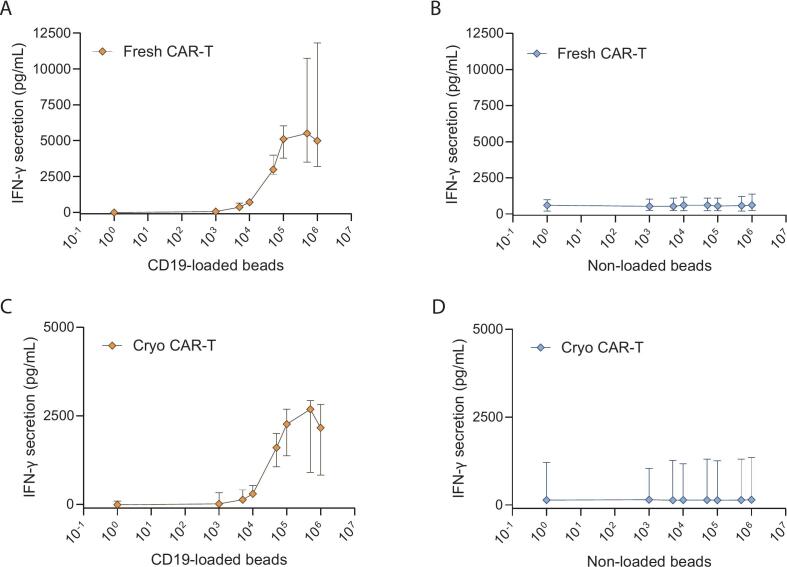
Dose dependent titration curve of 50 k fresh or 50 k cryopreserved CD19 CAR-T cells co-cultured with CD19-loaded or non-loaded beads. (A) Titration curve of 50 k fresh CD19 CAR-T cells generated from patients' apheresis material co-cultured with 500 k CD19-loaded beads. Median ± IQR is shown (*n* = 13 patients). (B) Titration curve of 50 k fresh CD19 CAR-T cells generated from patients' apheresis material co-cultured with 500 k non-loaded beads. Median ± IQR is shown (*n* = 13 patients). (C) Titration curve of 50 k cryopreserved CD19 CAR-T cells generated from patients' apheresis material co-cultured with 500 k CD19-loaded beads. Median ± IQR is shown (*n* = 5 patients). (D) Titration curve of 50 k cryopreserved CD19 CAR-T cells generated from patients' apheresis material co-cultured with 500 k non-loaded beads. Median ± IQR is shown (*n* = 5 patients). The wide IQR indicates large patient-to-patient variability in the potency test result.

To establish a statistically substantiated and clinically relevant potency threshold for fresh as well as cryopreserved CD19 CAR-T cells, an increasing amount of antigen-loaded beads (10^3^–10^6^) were co-incubated with 50 k fresh (*n* = 13 patients) or 50 k cryopreserved (*n* = 5 patients) CAR-T cells, which yielded a dose-dependent titration curve (Fig. 6). Although fresh CAR-T cells were more potent than cryopreserved CAR-T cells, clear dose-dependent increase in antigen-specific activation was observed for both drug products, with no significant activation by non-loaded beads, even at the highest concentration tested (Fig. 7). Notably, the wide IQR indicates large patient-to-patient variability in the potency test result. For antigen-specific activation, the concentration of IFN-γ was maximal at 500 k antigen-loaded beads, with a slight overall decrease in IFN-γ secretion detected upon stimulation with 1 million beads (Fig. 6, Fig. 7). Such a decrease could be due to suboptimal cross-linking of CARs due to excess antigen-loaded beads similar to the postzone phenomenon of the Hook effect (Namburi et al., 2014). These results further substantiated the selected amount of 500 k antigen-loaded beads for the assay, ensuring optimal and maximal fresh and cryopreserved CAR-T cell activation during each potency test.

The criterion to determine the potency threshold was defined as the first observed statistically significant increase in IFN-γ secretion where all tested CAR-T cell batches were activated compared to non-loaded beads as the negative control. Notably, although the first statistically significant (*P* = 0.002) increase in IFN-γ secretion (median: 47 pg/mL) was observed at the lowest amount of antigen-loaded beads tested, 3 out of 18 of the CAR-T cell batches were not activated at this bead concentration. Therefore, the second statistically significant (*P* < 0.001) increase in IFN-γ secretion was selected as the potency threshold since all CAR-T cell batches were activated compared to non-loaded bead stimulation, which corresponded to a potency threshold acceptance criterion of 313 pg/mL (median).

All fresh and cryopreserved CAR-T cell batches tested during the dose-dependent titration curves were considered fully functional and potent since significant antigen-specific activation was observed (Fig. 7). The median and range of IFN-γ secretion at the standardized 500 k antigen-loaded beads condition was 4992 pg/mL and 429–15,905 pg/mL, respectively. Therefore, the established potency threshold of 313 pg/mL was considered suitable to be implemented as the potency acceptance criterion for batch release of both fresh and cryopreserved CD19 CAR-T cell drug products, demonstrating that the assay complied with all validation acceptance criteria (Table 1).

Taken together, the optimized potency assay was validated and extensively characterized for routine GMP QC release testing of fresh and cryopreserved CD19 CAR-T cell drug products. Furthermore, the CD7 and HER2 potency assays were qualified and scientifically sound for CD7 and HER2 CAR-T cell potency testing during the preclinical and early development phase before GMP tech transfer, demonstrating proof-of-concept of the versatility of the assay. For GMP QC release testing, these two assays should be further characterized, optimized, and validated, as performed for the CD19 CAR-T cell potency assay. Our potency assay protocol and validation strategy underscore the importance of assay characterization, optimization, and standardization as well as sample characteristics of the tested CAR-T cell product during potency testing.

## 4 Conclusion

We here present a novel, versatile, fully standardized, and GMP-compliant bead-based potency assay suitable for routine GMP QC release testing of fresh and cryopreserved CAR-T cell products. The potency assay does not require cell lines expressing target antigens, thereby, eliminating the selection, procurement, and culture of cell lines for potency testing and strongly reducing labor burden. Furthermore, due to assay standardization, inherent analytical variation of cell-based assays within and between laboratories as a result of cell line heterogeneity are fully eliminated. Therefore, this bead-based potency assay is considered especially relevant for developers and manufacturers of multiple CAR-T cell products as well as decentralized manufacturers involved in CAR-T cell product comparability studies. Our assay protocol and validation strategy provides guidance for the development of a facile potency assay for CAR-T cells targeting any antigen of interest.

## CRediT authorship contribution statement

**Robin Dennebos:** Writing – review & editing, Writing – original draft, Visualization, Validation, Software, Project administration, Methodology, Investigation, Formal analysis, Data curation. **Macarena González-Corrales:** Writing – original draft, Visualization, Validation, Project administration, Methodology, Investigation, Formal analysis, Data curation. **Maria Lysandrou:** Writing – original draft, Validation, Project administration, Methodology, Investigation, Formal analysis, Data curation. **Nienke A.M. Smit:** Methodology, Investigation, Formal analysis, Data curation. **Yuzhu Qi:** Project administration, Methodology, Investigation, Formal analysis, Data curation. **Tom van Meerten:** Supervision, Resources, Investigation, Funding acquisition. **Jos G.W. Kosterink:** Supervision, Resources. **Gerwin Huls:** Supervision, Resources. **Harm-Jan Lourens:** Validation, Supervision, Resources, Project administration. **Bahez Gareb:** Writing – review & editing, Writing – original draft, Validation, Supervision, Project administration, Formal analysis, Conceptualization. **Edwin Bremer:** Writing – review & editing, Writing – original draft, Visualization, Validation, Supervision, Resources, Methodology, Investigation, Funding acquisition, Formal analysis, Conceptualization.

## Declaration of competing interest

All authors report no declaration of interests.

## Data Availability

Data will be made available on request.
